# Application of PEEP using the i-gel during volume-controlled ventilation in anesthetized, paralyzed patients

**DOI:** 10.1007/s00540-013-1628-2

**Published:** 2013-05-07

**Authors:** Yong Beom Kim, Young Jin Chang, Wol Seon Jung, Sang Ho Byen, Youn Yi Jo

**Affiliations:** Department of Anesthesiology and Pain Medicine, Gachon University Gil Medical Center, 1198 Guwol-dong, Namdong-gu, Incheon, 405-760 Korea

**Keywords:** i-gel, PEEP, Controlled ventilation

## Abstract

**Purpose:**

This prospective, randomized trial was designed to assess whether the i-gel supraglottic airway device is suitable for volume-controlled ventilation while applying positive end-expiratory pressure (PEEP) of 5 cmH_2_O under general anesthesia. It was believed that this device might improve arterial oxygenation.

**Methods:**

Forty adult patients (aged 20–60 years) scheduled for elective orthopedic surgery were enrolled in this study. Twenty patients were ventilated without external PEEP [zero positive end-expiratory pressure (ZEEP) group], and the other 20 were ventilated with PEEP 5 cmH_2_O (PEEP group) after placing an i-gel device. Volume-controlled ventilation at a tidal volume (TV) of 8 ml/kg of ideal body weight, leak volume, and arterial blood gas analysis were investigated.

**Results:**

The incidences of a significant leak were similar in the ZEEP and PEEP groups (3/20 and 1/20, respectively; *P* = 0.605), as were leak volumes. No significant PaO_2_ difference was observed between the two groups at 1 h after satisfactory i-gel insertion (215 ± 38 vs. 222 ± 54; *P* = 0.502).

**Conclusions:**

The use of an i-gel during PEEP application at 5 cmH_2_O did not increase the incidence of a significant air leak, and a PEEP of 5 cmH_2_O failed to improve arterial oxygenation during controlled ventilation in healthy adult patients.

## Introduction

The i-gel (Intersurgical, Wokingham, Berkshire, UK) was introduced into clinical practice in 2007. It is made of a thermoplastic elastomer, a soft gel-like substance [1]. This disposable device is designed to fit the perilaryngeal and hypopharyngeal structures without the use of an inflatable cuff, in contrast to other supraglottic airway devices [2], and has the advantages of easier insertion, minimal tissue compression, and fewer positional changes after cuff inflation.

The gold standard for airway management remains endotracheal intubation, but in view of the fact that minimizing interruptions to chest compressions, to maximize coronary and cerebral perfusion pressure, is the most important aspect of resuscitation, supraglottic airway devices could be a good substitute for airway management during cardiopulmonary resuscitation. Furthermore, i-gel insertion has been reported to be faster than ProSeal laryngeal mask airway (LMA), tracheal tube, and classic LMA during resuscitation [3].

Because most supraglottic airway devices, including i-gel, have a low airway leak pressure, moderate tidal volumes of 6–8 ml/kg have been recommended during positive pressure ventilation [4]. Although there were no differences in the amount of atelectasis in the patients undergoing general anesthesia without lung injury between a tidal volume of 10 and 6 ml/kg [5], in patients with acute lung injury, changes in tidal volume from 10 to 6 ml/kg increase the alveolar collapse, which can reversed by positive end-expiratory pressure (PEEP) application [6]. Applying PEEP in addition to controlled ventilation has been suggested to increase functional residual capacity and alveolar recruitment and to improve oxygenation and ventilation/perfusion mismatching under endotracheal intubation or when a ProSeal LMA is used [7–9]. However, because of the absence of an inflatable cuff, theoretically the i-gel might be more likely to have gas leaks during positive pressure ventilation than other supraglottic airway devices [10]. Recent studies support the use of i-gel during anesthesia for spontaneous breathing or controlled ventilation [10–12]. However, no study has yet shown that i-gel provides a good seal during PEEP application. Accordingly, the present study was designed to assess whether the i-gel is suitable for volume-controlled ventilation during PEEP application at 5 cmH_2_O under general anesthesia, and whether this device improves arterial oxygenation.

## Methods

This study was approved by our institutional review board, and written informed consent was obtained from all eligible participants.

### Subjects

Forty adult patients (aged 20–60 years) of American Society of Anesthesiologists (ASA) physical status I or II scheduled for elective orthopedic surgeries were enrolled in this study.

The following exclusion criteria were applied: a morbidly obese status (body mass index >30 kg/m^2^), a history of cerebrovascular or respiratory disease, neck or upper respiratory tract pathology, potentially difficult intubation, an increased risk of aspiration, and pregnancy. Patients were randomly allocated to one of two groups before the induction of anesthesia using computer-generated random numbers. The members of the ZEEP group were ventilated with no external PEEP application (*n* = 20), whereas members of the PEEP group were ventilated at a pressure of 5 cmH_2_O (*n* = 20) after placing the i-gel.

### Anesthesia

All 40 patients received intramuscular midazolam (0.05 mg/kg) and glycopyrrolate (0.2 mg) as premedication 1 h before anesthesia induction. On arrival at the operating room, standard monitors, including an electrocardiogram, pulse oximeter, and a noninvasive arterial pressure, were applied to all patients. i-gel sizes were selected using patient weight, as follows: size #3 for patients <50 kg, size #4 for patients between 50 and 70 kg, and size #5 for patients >70 kg. Anesthesia was induced with propofol 1.5–2.0 mg/kg, rocuronium 0.3 mg/kg, and alfentanil 10 μg/kg. After i-gel insertion, anesthesia was maintained with desflurane to maintain a bispectral index (BIS) between 40 and 50. Adequate placement was determined by observing the end-tidal carbon dioxide (ETCO_2_) waveform and chest movements, as previously reported [10]. If ventilation was inadequate, the device was gently pushed or the patient’s head and neck were repositioned. A failed attempt was defined as removal of the device from the mouth for reinsertion and was excluded from the statistic analysis. All patients were ventilated using an S/5 Avance anesthetic machine (GE Healthcare, Madison, WI, USA). After anesthesia induction, volume-controlled ventilation at a constant flow and an I:E ratio of 1:2 was performed at a tidal volume (TV) of 8 ml/kg of ideal body weight. Values were estimated using the following equation: 50 + 0.91 × (height in cm − 152.4) for men and 45.5 + 0.91 × (height in cm − 152.4) for women. A respiratory rate of 8–16 breaths/min was adjusted to maintain an ETCO_2_ of 30–35 mmHg at 40 % inspired oxygen in air using a fresh flow gas rate of 3 l/min.

### Evaluating leak volume and leak fraction

Hemodynamic variables, ETCO_2_, and ventilatory parameters were monitored and recorded at 5 min (T_1_, baseline values), 30 min (T_2_), and 1 h (T_3_) after satisfactory i-gel insertion. An arterial blood gas sample was obtained by a single sterile puncture from a radial artery at 1 h after i-gel insertion. Leak volume was defined as the difference between inspired tidal volume and expired tidal volume, and leak fraction (LF) was defined as leak volume/inspired tidal volume. The primary outcome variable was LF. The sample size was calculated on the basis of a preliminary study of 10 patients. The mean LFs were 0.05 and 0.08 before and after application of PEEP 5 cmH_2_O, and to detect a mean difference ± SD in actual LF of 0.03 ± 0.03 with an α-error of 0.05 and power of 80 % between the two groups in terms of applying PEEP, 16 patients were required in each group. Assuming a dropout rate of ~20 %, we calculated that 20 patients would be required per group.

### Statistical analysis

The statistical analysis was performed using SPSS ver. 12.0 (SPSS, Chicago, IL, USA). Results are expressed as mean ± SD or as numbers of patients. Differences between the two groups were analyzed using the *t* test. Statistical significance was accepted for *P* values <0.05.

## Results

All 40 patients enrolled and had their measures completed without any events. Patient characteristics and perioperative data were similar in the ZEEP and PEEP groups (Table 1). Selected i-gel sizes were nonsignificantly different in the two groups (*P* = 0.071). All i-gel devices were inserted at first attempt and an acceptable airway was achieved in all patients.

**Table 1:** Patient characteristics and perioperative data

	ZEEP (*n* = 20)	PEEP (*n* = 20)
Age (years)	44 ± 13	41 ± 14
Males/females	12/8	10/10
Weight (kg)	65 ± 11	65 ± 11
Height (cm)	168 ± 9	166 ± 8
Anesthesia time (min)	81 ± 40	85 ± 26
Operation time (min)	50 ± 41	57 ± 24
i-gel number #3/4/5	3/16/1	9/9/2

Data are presented as mean ± SD or numbers of patients

*ZEEP* zero positive end-expiratory pressure

*PEEP* 5 cmH_2_O positive end-expiratory pressure

The incidence of significant leaks (defined as an LF > 0.2) was 3/20 in the ZEEP group and 1/20 in the PEEP group, which was not a significant difference (*P* = 0.605). Actual leak fractions were similar between groups at T_1_ (0.08 ± 0.1 vs. 0.06 ± 0.07; *P* = 0.408), T_2_ (0.09 ± 0.09 vs. 0.05 ± 0.04; *P* = 0.135), and T_3_ (0.09 ± 0.08 vs. 0.05 ± 0.03; *P* = 0.208). Changes in respiratory parameters and arterial blood gas analysis results are presented in Table 2. Leak volumes were similar in the two groups. However, respiratory rate was significantly higher in the ZEEP group (at T_1_, T_2_, and T_3_; *P* = 0.05, 0.005, and 0.012, respectively). No significant intergroup difference was found for peak airway pressure, but mean airway pressure was significantly higher in the PEEP group at T_1_ (7 ± 1 vs. 9 ± 1, *P* < 0.001), T_2_ (7 ± 1 vs. 9 ± 1, *P* < 0.001), and T_3_ (7 ± 1 vs. 9 ± 1, *P* = 0.002). Air entry into the stomach was not detected by auscultation over the epigastric area in any patient. Mean PaO_2_ values in the ZEEP and PEEP groups at T_3_ were similar (215 ± 38 vs. 222 ± 54; *P* = 0.502). No patient experienced desaturation or CO_2_ retention gastric insufflation, regurgitation, or aspiration during surgery. Finally, no blood was visible on i-gels after removal, and no patient complained of a severe sore throat or soft tissue or tooth injury.

**Table 2:** Perioperative respiratory and blood gas parameters

	ZEEP (*n* = 20)	PEEP (*n* = 20)	*P* value
Leak volume (ml/kg)			
T_1_	39 ± 47	30 ± 37	0.515
T_2_	45 ± 41	30 ± 19	0.226
T_3_	47 ± 42	27 ± 19	0.063
Leak fraction			
T_1_	0.08 ± 0.1	0.06 ± 0.07	0.408
T_2_	0.09 ± 0.09	0.05 ± 0.04	0.135
T_3_	0.09 ± 0.08	0.05 ± 0.03	0.208
Peak airway pressure (cmH_2_O)			
T_1_	14 ± 3	16 ± 3	0.086
T_2_	16 ± 4	17 ± 3	0.412
T_3_	16 ± 4	18 ± 3	0.209
Mean airway pressure (cmH_2_O)			
T_1_	7 ± 1	9 ± 1*	<0.001
T_2_	7 ± 1	9 ± 1*	<0.001
T_3_	7 ± 1	9 ± 1*	0.002
Expiratory tidal volume (ml)			
T_1_	479 ± 92	504 ± 92	0.877
T_2_	480 ± 97	466 ± 141	0.579
T_3_	479 ± 92	504 ± 92	0.412
Respiratory rate (breaths/min)			
T_1_	13 ± 2	12 ± 2*	0.015
T_2_	13 ± 2	11 ± 2*	0.005
T_3_	13 ± 2	11 ± 2*	0.012
ETCO_2_ (mmHg)			
T_1_	34 ± 2	33 ± 3	0.476
T_2_	33 ± 2	32 ± 2	0.213
T_3_	33 ± 2	32 ± 2	0.563
pH	7.47 ± 0.02	7.46 ± 0.05	0.246
PaO_2_ (mmHg)	215 ± 38	222 ± 54	0.631
PaCO_2_ (mmHg)	37 ± 2	36 ± 4	0.228

5 min (T_1_), 30 min (T_2_), and 1 h (T_3_) after proper insertion of the i-gel device. Data are presented as mean ± SD

*ZEEP* zero positive end-expiratory pressure

*PEEP* 5 cmH_2_O of positive end-expiratory pressure

* *P* < 0.05 vs. ZEEP group at each time point

## Discussion

We found that the use of an i-gel and the application of PEEP at 5 cmH_2_O did not increase significant air leak incidence or leak volume. Furthermore, the i-gel device provided acceptable airways without any desaturation or CO_2_ retention event regardless of PEEP. In addition, PEEP at 5 cmH_2_O failed to improve arterial oxygenation during controlled ventilation using an i-gel.

I-gel is a novel supraglottic airway device with a shorter insertion time, higher mean leak pressure, and a better fiberoptic view score than the standard disposable laryngeal mask airway, and has been reported to reduce the incidence of sore throat, dysphagia, and neck pain [13]. In another study that compared the LMA Supreme and the i-gel in paralyzed, ventilated patients undergoing gynecological laparoscopic procedures [11], similar satisfactory results were obtained for ease of insertion, success rate on first attempt, time to insertion, and oropharyngeal leak pressure. Gatward et al. [3] reported that time taken for i-gel insertion was ~50 % that of other airway devices, such as the tracheal tube classic LMA and ProSeal LMA during chest compression for cardiopulmonary resuscitation. We believe that the use of i-gel with proper PEEP could contribute to adequate ventilation and prevent atelectasis during general anesthesia or resuscitation.

Because, theoretically, the absence of an inflatable cuff might increase the risk of gas leaks during the application of PEEP, the objective of this study was to determined whether the application of PEEP with an i-gel fitted could maintain acceptable ventilation without clinically significant air leakage. Uppal et al. [10] reported that the i-gel achieved a median airway leak pressure of 28 cmH_2_O, which is higher than that of conventional LMA [22 ± 6 (8–40) cmH_2_O] and similar to that of the ProSeal LMA [27 ± 7 (10–40) cmH_2_O] [14]. Although peak airway pressure was slightly higher in the PEEP group (not significant), it might not exceed the leak pressure. Considering that peak airway pressure was 10–23 cmH_2_O and mean airway pressure was 6–11 cmH_2_O in both our study groups, it was not unexpected that we did not encounter any clinically eventful oropharyngeal air leakage.

The use of high fraction inspired oxygen during anesthetic induction causes atelectasis to develop within minutes. However, the application of adequate PEEP can prevent atelectasis and provide a higher level of oxygenation [15]. Goldmann et al. [8] demonstrated that the application of PEEP 5 cmH_2_O under general anesthesia improved gas exchange in children when the ProSeal LMA was used with an inflatable cuff. However, improvements in functional residual capacity do not always correlate with major changes in oxygenation. For example, Futier et al. [16] reported, in nonobese patients, that PEEP at 5 cmH_2_O improved end-expiratory lung volume 15 % but not oxygenation. Although gas exchange is often used to assess lung function during controlled ventilation, it has been reported that oxygenation does not provide a specific measure of nonaerating lung tissue [17, 18]. In a study of anatomical shunt compartment by whole lung computed tomography and of functional shunt using blood gas analysis at PEEP values of 5 and 15 cmH_2_O, functional shunt was found to be poorly correlated with the anatomical shunt compartment (*r* ^2^ = 0.174), which was attributed to a large variability in apparent perfusion ratio [18]. It was concluded that gas exchange variations could not be used with sufficient confidence to assess anatomical lung recruitment [18]. In the present study, we did not detect improvements in arterial oxygenation, and thus we suggest that further study using computed tomography (CT) or imaging devices be conducted to determine the effect of PEEP on atelectasis and alveolar recruitment.

The present study has several limitations. First, leak pressure was not measured using a manometer; instead, we checked for the absence of any audible throat noise and the proper achievement of tidal volume because we wanted the study to reflect clinical usability. Also, we did not confirm the proper positioning using fiberoptic bronchoscopy. However, we ascertained that ventilation was not compromised in any patient. Second, morbidly obese patients and those with a respiratory problem, an increased risk of aspiration, or pregnant status were excluded, and thus our results cannot be directly applied to patients with reduced respiratory compliance.

We conclude that the use of an i-gel and the application of PEEP at 5 cmH_2_O did not increase the incidence of a significant air leak. However, a PEEP of 5 cmH_2_O failed to improve arterial oxygenation during controlled ventilation with the i-gel device in healthy adult patients.
